# Negligible import of enteric pathogens by newly arrived asylum seekers and no impact on incidence of notified *Salmonella* and *Shigella* infections and outbreaks in Rhineland-Palatinate, Germany, January 2015 to May 2016

**DOI:** 10.2807/1560-7917.ES.2018.23.20.17-00463

**Published:** 2018-05-17

**Authors:** Lutz Ehlkes, Maja George, Donald Knautz, Florian Burckhardt, Klaus Jahn, Manfred Vogt, Philipp Zanger

**Affiliations:** 1 Federal State Agency for Consumer and Health Protection Rhineland-Palatinate, Koblenz, Germany; 2 Postgraduate Training for Applied Epidemiology (PAE), Robert Koch Institute, Berlin, Germany; 3 European Programme for Intervention Epidemiology Training (EPIET), European Centre for Disease Prevention and Control (ECDC), Stockholm, Sweden; 4 These authors contributed equally to this work and share first authorship; 5 Federal State Ministry for Social Affairs, Employment, Health, and Demographics Rhineland-Palatinate, Mainz, Germany; 6 Heidelberg Institute of Public Health, University Hospitals, Heidelberg, Germany; 7 Department of Infectious Diseases, Medical Microbiology and Hygiene, University Hospitals, Heidelberg, Germany

**Keywords:** (MESH) Human migration, Refugees, Mass Screening, Enterobacteriaceae, Helminthiasis, Schistosomiasis

## Abstract

The 2015 refugee crisis raised concerns about an import of infectious diseases affecting the German population. **Aims:** To evaluate public and individual health benefits of stool screening, and explore whether importation of enteric pathogens by newly arrived asylum seekers impacts on the host population. **Methods**: We used data from mandatory stool screening to determine the overall, age, sex, and country-specific prevalence of enteric bacteria and helminths. We used surveillance data to assess whether the number of incoming asylum seekers influenced notifications of salmonellosis and shigellosis in Rhineland-Palatinate. **Results**: *Salmonella* were found in 0.2% (95% confidence interval (CI) 0.2–0.3%) of 23,410 samples collected from January 2015 to May 2016. Prevalence was highest in children under 5 years (0.8%; 95% CI: 0.5–1.3%). No *Shigella* or invasive *Salmonella* spp. were detected. In a subset of 14,511 samples, the prevalence of helminth infestation was 2.4% (95% CI: 2.1–2.6%), with highest proportions detected in adolescents (4.6%; 95% CI 3.8–5.4%) and among Eritreans (9.3%; 95% CI: 7.0–12.0%); in the latter particularly *Schistosoma mansoni* and *Taenia* spp. The increase in asylum applications did not increase notifications of salmonellosis and shigellosis. No transmission from asylum seekers to German residents was notified. **Conclusion**: Public health risk associated with imported enteric pathogens is very low overall. Addressing individual and public health risks, we recommend replacing stool screening of all newly arrived asylum seekers by a targeted approach, with target groups and approaches being adapted if necessary. Target groups supported by our data are children, adolescents, and Eritreans.

## Introduction

Sparked primarily by the Syrian civil war but also by other conflicts in the Middle East and Southern Asia, the number of first-time asylum applications in Germany increased more than fourfold in 2 years [1]. In response, Rhineland-Palatinate, a federal state with a population of ca 4 million, established 29 asylum seeker reception centres, temporarily accommodating over 60,000 asylum seekers.

A large proportion of these asylum seekers, i.e. displaced people whose refugee status has yet to be confirmed, originate from Eastern Africa, and Western and Southern Asia (regions according to [2]). There, the standard of water, sanitation, and food hygiene is lower than in Western Europe [3], resulting in a higher incidence of gastro-intestinal infections. Besides, migration itself often entails exposure to unsafe water and food. Such reasoning underlines the hypothesis of migration fostering importation of enteric pathogens to Europe, with a potential of onward transmission as demonstrated by outbreaks in asylum seeker reception centres [4]. These fears are contrasted by expert opinion on imported infections remaining largely confined to the migrant population and thus being negligible for European public health [5]. To date; however, little data are available to support this view for enteric pathogens.

National and federal-state law regulates the health status assessment of newly arrived asylum seekers in Rhineland-Palatinate [6,7]. Upon first presentation at an asylum seeker reception centre, each individual undergoes a mandatory medical examination. Apart from screening for tuberculosis, this examination includes a screening of a single stool sample for *Salmonella* spp., *Shigella* spp., and helminth eggs. To date, there has been no public health evaluation of stool screening in newly arrived asylum seekers in Rhineland-Palatinate, and publications regarding the results and public health benefits of similar screening programs from other federal states in Germany and European countries are limited [8].

The aim of this study was to evaluate the public and individual health benefits of stool screening, and to explore whether the import of enteric pathogens by asylum seekers impacts on the host population’s health. To this end, we describe the prevalence of enteric pathogens among incoming asylum seekers and stratify by age, sex, and geographic origin as potential risk factors for pathogen carriage. Additionally, by using data from the federal state-wide mandatory notification system, we check whether the number of incoming asylum seekers influenced the number of notified cases and outbreaks of salmonellosis and shigellosis in Rhineland-Palatinate.

## Methods

This study analyses data from mandatory stool screening collected from January 2015 to May 2016. Laboratory analyses were performed by the Federal State Agency for Consumer and Health Protection Rhineland Palatinate. Stool samples were tested for *Salmonella* spp. and *Shigella* spp. During the peak period of migration, microscopy for helminths was conducted in two thirds of samples only. This was achieved by rotating the subset of reception centres included in the study every 2 weeks.

### Laboratory methods

Deoxycholate-Citrate (DC) and Xylose-Lysine-Deoxycholate (XLD) agar plates as well as Tetrathionat and Selenit enrichment bouillons (all Oxoid, Wesel, Germany) were inoculated with stool and incubated overnight at either 37 °C or, in case of Tetrathionat bouillon, at 42 °C. Suspicious colonies were classified as *Salmonella* spp. based on the results from decarboxylase activity, hydrogen sulphide and indole production, and mannitol fermentation. Putative *Salmonella* spp. and *Shigella* spp. were further serotyped by agglutination (Sifin, Berlin, Germany). Cellophane thick smears were prepared for stool microscopy as proposed by Kato 1954 [9]. All diagnostic parameters presented in this study are subject to a quality management system which includes regular participation in ring trials.

### Data management and analysis

Date of birth, sex, country of origin, and laboratory results were extracted from the electronic laboratory information and management system (Blomesystem GmbH, Jena, Germany), and imported into Stata 14 while omitting personal identifiers. We calculated prevalences of enteric pathogens by age group (0–4, 5–9, 10–19, 20–29, 30–39, 40–49, ≥ 50 years) and country of origin. Countries were classified into geographic regions according to the M49 standard used by the United Nations Statistics Division [2]. In order to identify predictors of pathogen carriage, age group- and sex-specific prevalences were compared using Pearson’s chi-squared test. When calculating prevalences of enteric pathogens in the overall study group, the variance estimate was corrected for clustering of subjects on the country level using the ‘cluster’ option of the ‘proportion’ command in Stata 14.

Detection of *Salmonella* spp. (invasive and non-invasive) and *Shigella* spp., but not of the helminths discussed in this study, is notifiable according to §7 of the German Protection against Infection Act (Infektionsschutzgesetz, IfSG) [7]. For the period from 2007 to 2016, the yearly numbers of salmonellosis and shigellosis notifications and outbreaks in Rhineland-Palatinate were extracted from the German Infectious Disease Surveillance Network Database (SurvNet) and compared with the published number of asylum applications in Rhineland-Palatinate [1]. In October 2015, the asylum status of cases was added to mandatory notifications in SurvNet. We extracted notified cases of salmonellosis and shigellosis in newly arrived asylum seekers from SurvNet for the period from October 2015 to December 2016 and checked whether these were linked to secondary cases, which would be notifiable according to §6 IfSG [7].

### Ethics

Stool screening as described is mandated by the IfSG [7] and the Administrative Act number 21260 of the federal state of Rhineland-Palatinate [6]. Thus, no individual informed consent was sought for this research.

## Results

In total, 23,410 stool samples were tested from January 2015 to May 2016 (Table 1). The majority of samples were from newly arrived asylum seekers from Syria (n = 8,128), followed by Afghanistan (n = 3,903), Albania (n = 3,196), and Kosovo (n = 1,748). The median age was 22 years (range: 0–90; interquartile range (IQR): 0–87). Information on sex was available for 18,720 samples, 7,307 were from females (39.0%).

**Table 1:** Enteric bacteria in newly arrived asylum seekers detected through stool screening, by country and region of origin, Rhineland-Palatinate, Germany, January 2015–May 2016 (n = 23,410)

Region^a^ and country of origin	Total samples	Culture results					
		*Salmonella* spp.			*Shigella* spp.		
		n	%	95% CI	n	%	97.5% CI^b^
**Southern Europe**							
Albania	3,196	5	0.2	0.0–0.4	0	0.0	0.0–0.1
Kosovo*	1,748	3	0.2	0.0–0.1	0	0.0	0.0–0.2
Serbia	831	0	0.0	0.0–0.0^b^	0	0.0	0.0–0.4
the former Yugoslav Republic of Macedonia	586	2	0.3	0.0–1.2	0	0.0	0.0–0.6
Bosnia-Herzegovina	284	0	0.0	0.0–1.3^b^	0	0.0	0.0–1.3
**Subtotal**	**6,645**	**10**	**0.2**	**0.1–0.3**	**0**	**0.0**	**0.0–0.1**
**Eastern Africa**							
Eritrea	596	2	0.3	0.0–1.2	0	0.0	0.0–0.6
Somalia	419	0	0.0	0.0–0.9^b^	0	0.0	0.0–0.9
Other^c^	12	0	0.0	0.0–26.5^b^	0	0.0	0.0–26.5
**Subtotal**	**1,027**	**2**	**0.2**	**0.0–0.7**	**0**	**0.0**	**0.0–0.4**
**Western Asia**							
Syria	8,128	23	0.3	0.2–0.4	0	0.0	0.0–0.1
Armenia	218	0	0.0	0.0–1.7^b^	0	0.0	0.0–1.7
Iraq	120	0	0.0	0.0–3.0^b^	0	0.0	0.0–0.0
Other^c^	151	0	0.0	0.0–2.4^b^	0	0.0	0.0–2.4
**Subtotal**	**8,617**	**23**	**0.3**	**0.2–0.4**	**0**	**0.0**	**0.0–0.0**
**Southern Asia**							
Afghanistan	3,903	11	0.3	0.1–0.5	0	0.0	0.0–0.1
Pakistan	787	1	0.1	0.0–0.7	0	0.0	0.0–0.5
Iran	530	0	0.0	0.0–0.7^b^	0	0.0	0.0–0.7
Other^c^	16	0	0.0	0.0–20.6^b^	0	0.0	0.0–20.6
**Subtotal**	**5,236**	**12**	**0.2**	**0.1–0.4**	**0**	**0.0**	**0.0–0.1**
**Northern Africa**							
Egypt	121	1	0.8	0.0–4.5	0	0.0	0.0–3.0
Other^c^	37	1^e^	2.7	0.1–14.2	0	0.0	0.0–9.5
**Subtotal**	**158**	**2**	**1.3**	**0.2–4.5**	**0**	**0.0**	**0.0–2.3**
Other^d^	246	1^f^	0.4	0.0–2.2	0	0.0	0.0–1.5
Unknown	1,481	2	0.1	0.0–0.1	0	0.0	0.0–0.3
**Total**	**23,410**	**52**	**0.2**	**0.2–0.3^g^**	**0**	**0.0**	**0.0–0.2^g^**

CI: confidence interval

^a^ Grouping of regions according to [2], except for Kosovo*.

^b^ Zero percent estimates are provided with a one-sided, 97.5% CI.

^c^ Combines countries from given region with fewer than 100 subjects examined.

^d^ Combines countries from regions not displayed in table and with fewer than 100 subjects examined.

^e^ Country of origin of this sample: Morocco (1/16).

^f^ Country of origin of this sample: Nigeria (1/13).

^g^ Variance estimate was calculated while accounting for clustering of subjects by country.

*This designation is without prejudice to positions on status, and is in line with UNSCR 1244/1999 and the ICJ Opinion on the Kosovo declaration of independence.

### Enterobacteria

Fifty-two of the 23,410 samples tested positive for *Salmonella* spp. (0.2%).

The highest prevalence of *Salmonella* spp. in stools was detected in asylum seekers from Egypt (0.8%), followed by Afghanistan, Eritrea, the former Yugoslav Republic of Macedonia, and Syria (all 0.3%). No *Salmonella* were detected in samples from Armenia, Bosnia-Herzegovina, Iran, Iraq, Serbia, or Somalia. *Salmonella* spp. were more commonly identified in stools from children under 10 years of age when compared with all other age groups (0.6% vs 0.1%; chi-squared p < 0.001) (Table 2). Females were more likely to carry *Salmonella* spp. (n = 29/7,307; 0.4%; 95% CI: 0.3–0.6) than males (n = 16/11,413; 0.1%; 95% CI: 0.1–0.2; chi-squared p < 0.001).

**Table 2:** Enteric bacteria and helminths in newly arrived asylum seekers detected through stool screening, by age, Rhineland-Palatinate, Germany, January 2015– May 2016 (n = 23,410 screened for enteric bacteria; n = 14,511 screened for helminth eggs)

Age group (years)	Enteric bacteria				Helminth eggs			
	Positive		Negative		Positive		Negative	
	n	%	n	%	n	%	n	%
0 to < 5	20	0.8	2,391	99.2	19	0.9	2,049	99.1
5 to < 10	6	0.3	2,060	99.7	36	2.7	1,306	97.3
10 to < 20	7	0.1	5,178	99.9	134	4.6	2,805	95.4
20 to < 30	12	0.2	7,150	99.8	105	2.4	4,309	97.6
30 to < 40	4	0.1	3,897	99.9	37	1.6	2,242	98.4
40 to < 50	2	0.1	1,781	99.9	9	0.9	967	99.1
≥ 50	1	0.1	901	99.9	4	0.8	489	99.2
**Total**	**52**	**0.2**	**23,358**	**99.8**	**344**	**2.4**	**14,167**	**97.6**

In the 52 *Salmonella*-positive stool samples, we identified 53 different serovars (one sample with two serovars): *S*. Enteritidis (n = 10), *S*. Infantis (n = 6), *S*. Kentucky (n = 6), *S*. Typhimurium (n = 2), *S*. Typhimurium, monophasic variant (n = 3), *S*. Agona (n = 2), and *Salmonella* sp., non-typeable (n = 3), plus the following rare serovars in Germany: *S*. Strathcona (n = 2), *S*. Newport (n = 2), *S*. Abony (n = 1), *S*. Anatum (n = 1), *S.* Give (n = 1), *S*. Bareilly (n = 1), *S*. Bovismorbificans (n = 1), *S*. Braenderup (n = 1), *S*. Corvallis (n = 1), *S*. Hadar (n = 1), *S*. Kimuenza (n = 1), *S*. Kottbus (n = 1), *S*. Livingstone (n = 1), *S*. Manhattan (n = 1), *S*. Meleagridis (n = 1), *S*. Salford (n = 1), *S*. Reading (n = 1), *S*. Tennessee (n = 1), and *S*. Uganda (n = 1).

We did not detect entero-invasive *Salmonella* (*S.* Typhi, *S.* Paratyphi) or *Shigella* spp. in any of the samples.

### Helminths

Overall, 14,511 samples were tested for helminth eggs. The predominant country of origin for those newly arrived asylum seekers tested was Afghanistan (n = 3,200), followed by Syria (n = 2,628), Albania (n = 2,436), and Kosovo (n = 1,704). The median age was 21 years (range: 0–88; IQR 0–78). Sex was available for 11,597 samples, 4,356 were from females (37.6%).

A total of 344 samples tested positive (prevalence 2.4%; 95% CI: 2.1–2.6). Males (n = 205/7,241; 2.8%; 95% CI: 2.5–3.2) had a significantly higher prevalence of helminth infestation compared with females (n = 95/4,356; 2.2%: 95% CI: 1.8–2.7; chi-squared p = 0.033). Most commonly identified helminths were *Ascaris lumbricoides* (0.7%; 95% CI: 0.5–0.8) and *Trichuris trichiura* (0.6%; 95% CI: 0.4–0.7). *Hymenolepis nana* was detected in 49 samples (0.3%; 95% CI: 0.2–0.4), *Schistosoma mansoni* in 47 (0.3%; 95% CI: 0.2–0.4), *Enterobius vermicularis* in 36 (0.2%; 95% CI: 0.2–0.3), *hookworm* in 23 (0.2%; 95% CI: 0.1–0.2), and *Taenia* spp. in 12 (0.1%; 95% CI: 0.0–0.1) (Table 3). Larvae from *Strongyloides stercoralis* were seen in three samples.

**Table 3:** Helminth infestation in newly arrived asylum seekers detected by stool screening, Rhineland-Palatinate, Germany, January 2015–May 2016 (n = 14,511)

Region^a^ and country of origin	Sample size	Microscopy results															
		Overall positive		*Ascaris lumbricoides*		*Trichuris trichiura*		*Hymenolepis nana*		*Schistosoma mansoni*		*Enterobius vermicularis*		*Ancylostoma/Necator* spp.		*Taenia* spp.	
		n	%	n	%	n	%	n	%	n	%	n	%	n	%	n	%
**Southern Europe**																	
Albania	2,436	36	1.5	1	0.0	20	0.8	2	0.1	0	0.0	11	0.5	0	0.0	2	0.1
Kosovo*	1,704	11	0.6	0	0.0	6	0.4	1	0.1	0	0.0	4	0.2	0	0.0	0	0.0
Serbia	604	14	2.3	2	0.3	7	1.2	2	0.3	0	0.0	3	0.5	0	0.0	0	0.0
the former Yugoslav Republic of Macedonia	436	2	0.5	0	0.0	2	0.5	0	0.0	0	0.0	0	0.0	0	0.0	0	0.0
Bosnia-Herzegovina	180	1	0.6	0	0.0	0	0.0	1	0.6	0	0.0	0	0.0	0	0.0	0	0.0
**Subtotal**	**5,360**	**64**	**1.2**	**3**	**0.1**	**35**	**0.7**	**6**	**0.1**	**0**	**0.0**	**18**	**0.3**	**0**	**0.0**	**2**	**0.0**
**Eastern Africa**																	
Eritrea	571	53	9.3	1	0.2	1	0.2	4	0.7	40	7.0	0	0.0	2	0.4	5	0.9
Somalia	403	22	5.5	0	0.0	19	4.7	0	0.0	0	0.0	2	0.5	1	0.2	0	0.0
Other^b^	11	1	9.1	1	9.1	0	0.0	0	0.0	0	0.0	0	0.0	0	0.0	0	0.0
**Subtotal**	**985**	**76**	**7.7**	**2**	**0.2**	**20**	**2.0**	**4**	**0.4**	**40**	**4.1**	**2**	**0.2**	**3**	**0.3**	**5**	**0.5**
**Western Asia**																	
Syria	2,628	15	0.6	0	0.0	1	0.0	3	0.1	0	0.0	8	0.3	1	0.0	2	0.1
Armenia	199	0	0.0	0	0.0	0	0.0	0	0.0	0	0.0	0	0.0	0	0.0	0	0.0
Other^b^	174	2	1.1	0	0.0	0	0.0	1	0.6	0	0.0	0	0.0	0	0.0	1	0.6
**Subtotal**	**3,001**	**17**	**0.6**	**0**	**0.0**	**1**	**0.0**	**4**	**0.1**	**0**	**0.0**	**8**	**0.3**	**1**	**0.0**	**3**	**0.1**
**Southern Asia**																	
Afghanistan	3,200	133^d^	4.2	80	2.5	13	0.4	24	0.8	0	0.0	7	0.2	5	0.2	1	0.0
Pakistan	651	29	4.5	6	0.9	6	0.9	5	0.8	0	0.0	0	0.0	11	1.7	1	0.2
Iran	236	0	0.0	0	0.0	0	0.0	0	0.0	0	0.0	0	0.0	0	0.0	0	0.0
Other^b^	7	2	28.6	0	0.0	1	14.3	1	14.3	0	0.0	0	0.0	0	0.0	0	0.0
**Subtotal**	**4,094**	**164^d^**	**4.0**	**86**	**2.1**	**20**	**0.5**	**30**	**0.7**	**0**	**0.0**	**7**	**0.2**	**16**	**0.4**	**2**	**0.0**
Other^c^	287	7	2.4	0	0.0	1	0.3	0	0.0	4	1.4	0	0.0	2	0.7	0	0.0
Unknown	784	16	2.0	3	0.4	3	0.4	5	0.6	3	0.4	1	0.1	1	0.1	0	0.0
**Total**	**14,511**	**344**	**2.4**	**94**	**0.7**	**80**	**0.6**	**49**	**0.3**	**47**	**0.3**	**36**	**0.2**	**23**	**0.2**	**12**	**0.1**

Positive samples in the ‘other’ categories: *Ascaris lumbricoides*: Ethiopia (1/9); *Trichuris trichiura*: Bangladesh (1/6), Nigeria (1/13); *Hymenolepis nana*: Bangladesh (1/6), Irak (1/56); *Schistosoma mansoni*: Central African Republic (2/29), Egypt (1/90), Equatorial Guinea (n = 1/1); *Ancylostoma/Necator* spp.: Mali (1/7), Sierra Leone (1/1); *Taenia* sp: Georgia (1/75).

^a^ Grouping of regions according to [2], except for Kosovo.

^b^ Combines countries from given region with fewer than 100 subjects examined.

^c^ Combines countries with fewer than 100 subjects examined from regions not included in this table.

^d^ Includes three *Strongyloides stercoralis* positive samples not otherwise displayed in this table.

*This designation is without prejudice to positions on status, and is in line with United Nations Security Council Resolution 1244/99 and the International Court of Justice Opinion on the Kosovo declaration of independence.

Helminth infestation was most commonly detected in asylum seekers from Eastern Africa namely Eritrea (9.3%; 95% CI: 7.0–12.0; predominantly *S. mansoni*), and Somalia (5.5%; 95% CI: 3.5–8.1; predominantly *T. trichiura*), followed by the Southern Asian countries Pakistan (4.5%; 95% CI: 3.0–6.3; predominantly hookworm) and Afghanistan (4.2%; 95% CI: 3.5–4.9; predominantly *A. lumbricoides*). The prevalence of helminth infestation was more common in those under 20 years of age, compared with older age groups (3.0% vs 1.9%; chi-squared p < 0.001) (Table 2).

### Impact of asylum seekers on notified cases and outbreaks

While the number of asylum applications increased over 40-fold from 2007 to 2016, notifications and outbreaks of salmonellosis and shigellosis continuously decreased (Table 4).

**Table 4:** Number of asylum applications and salmonellosis and shigellosis notifications, incidence and outbreaks in Rhineland-Palatinate, Germany, January 2007–December 2016

Year	Asylum applications^a^	Salmonellosis notifications			Shigellosis notifications		
		Confirmed cases^b^	Incidence^c^	Number of outbreaks^d^	Confirmed cases^b^	Incidence^c^	Number of outbreak^d^
2007	767	3,635	90.6	298	44	1.1	5
2008	883	2,607	65.0	221	29	0.7	1
2009	1,106	1,691	42.1	125	40	1.0	4
2010	1,653	1,466	36.5	84	49	1.2	4
2011	1,830	1,287	32.1	67	45	1.1	2
2012	2,582	1,142	28.5	58	28	0.7	2
2013	4,383	943	23.5	42	59	1.5	3
2014	6,922	881	22.0	46	34	0.9	4
2015	17,625	726	18.1	26	24	0.6	2
2016	36,985	729	18.2	31	31	0.8	1

^a^ To the federal state of Rhineland-Palatinate [1].

^b^ Notified to the responsible health authorities according to §7 of the German Protection Against Infection Act (Infektionsschutzgesetz) [7].

^c^ Notified laboratory-confirmed cases per 100,000 population.

^d^ Defined as at least two cases with an epidemiological link; notified according to the responsible health authorities according to §6 German Protection Against Infection Act (Infektionsschutzgesetz) [7].

Since the introduction of asylum status to mandatory notifications in SurvNet in October 2015 until the end of 2016, four confirmed cases of salmonellosis and three confirmed cases of shigellosis were reported in asylum seekers in Rhineland-Palatinate, compared with 896 salmonellosis and 41 shigellosis cases in the host population. One of the notified salmonellosis cases in asylum seekers occurred secondary to a German case. There were no records of secondary transmission of *Salmonella* spp. or *Shigella* spp. from an asylum seeker to either the host population or other asylum seekers.

Two cases of *S.* Typhi/*S.* Paratyphi were notified in the resident population in Rhineland-Palatinate during this time period, both of whom reported prior travel to endemic countries (India and Bangladesh).

## Discussion

Our analysis of a large sample of screened stools and surveillance data provides evidence against the hypothesis that the import of enteric bacteria by newly arrived asylum seekers has an impact on public health of the host population. The increase in the number of asylum applications in Rhineland-Palatinate did not lead to an increase of notified cases and outbreaks of salmonellosis and shigellosis. Furthermore, we did not detect a single record of *Salmonella* spp. or *Shigella* spp. transmission event following a case in an asylum seeker. Only 0.2% of samples from newly arrived asylum seekers tested positive for *Salmonella* spp. and none for *Shigella* spp., corroborating the results of one report from Bavaria [8] and re-confirming that import of enteric bacteria by asylum seekers is rare.

We found helminth infestation in 2.4% of newly arrived asylums seekers. This is well above the prevalence reported in Germany, and other countries in Western and Northern Europe [10]. To adequately discuss the value of screening for helminths, these results need to be assessed in terms of (i) person-to-person transmissibility and (ii) morbidity in case of infection, with the latter being particularly important in infections with long latency periods and irreversible sequelae.

Many geohelminths require maturation in soil before they become infective (e.g. *A. lumbricoides and T. trichiura*) and, as in the case of *S. stercoralis* and hookworms *(Ancylostomatidae),* infect humans through penetration of healthy skin. These mechanisms put open defecation and barefoot walking at the centre of transmission, which grossly reduces the probability of transmission in Germany. Irreversible sequelae through low burden, asymptomatic infections are uncommon in the immunocompetent host, allowing for curative treatment with onset of symptoms. Hence, an increased prevalence of geohelminths in asylum seekers compared with the host population does not justify general screening.

We detected 47 cases of schistosomiasis, 40 of whom were newly arrived asylum seekers from Eritrea. Although human schistosomiasis finds most suitable ecologic conditions for its transmission in the tropics, a recent outbreak of *S. haematobium* infection in visitors to the Mediterranean island of Corsica demonstrates a low, but tangible risk of its emergence in Europe [11]. Infection with schistosomes often remains asymptomatic for years, yet the tissue damage caused during this period remains irreversible [12], rendering early diagnosis through screening of asymptomatic individuals that were exposed at high-risk destinations important.

*E. vermicularis* was detected in 0.2% of newly arrived asylum seekers, predominantly in children. Enterobiasis is easily transmitted from person-to-person, common in autochthonous populations in Europe [10], and reported to cause outbreaks in childcare facilities in Germany [13]. It generally responds well to treatment and has no serious health effects. Therefore, we consider it to be unlikely that importation of *E. vermicularis* to an extent described here has a negative impact on public health in Germany or elsewhere in Europe.

We detected *H. nana* in 0.3% of samples (49/14,511), of which 34 were from children and adolescents. A study from Italy found *H. nana* in seven of 5,351 stool samples (0.1%) of hospitalised patients, six of whom were children under 15 years [14]. Hence, prevalence of hymenolepiasis in newly arrived asylum seekers appears somewhat higher when compared with the autochthonous population in Europe. Direct person-to-person transmission of *H. nana* via ingestion of eggs, including autoinfection, is common. Infestation with *H. nana* is more common among children, not associated with long-term sequelae, and responds well to treatment. Therefore, we conclude, that its importation could have some minor negative public health impact in Germany or elsewhere in Europe.

Apart from being the definitive host of *T. solium*, humans can also become infected by its eggs, which then develop into the parasitic stage that is usually seen in the intermediate host (i.e. in pigs). Cysticercosis is a rare, but disabling and potentially life-threatening disease, as the parasite can affect the central nervous system (neurocysticercosis) and lead to serious, incurable neurologic symptoms [15]. Standard microscopy does not allow differentiation between the eggs of *T. solium* and *T. saginata.* This circumstance complicates the risk assessment, as only eggs of *T. solium* infest humans. Information with regard to *T. solium* endemicity is sparse, and unavailable for Afghanistan, Albania, Eritrea, and Pakistan [16], i.e. those countries where nine of 12 *Taenia* spp. infections in our study population were imported from. Therefore, our data support a marginal chance for severe morbidity caused by secondary cysticercosis following import by newly arrived asylum seekers. This; however, will primarily affect asylum seekers themselves, through autoinfection, and must be balanced against occurrence of cysticercosis after import of *T. solium* eggs through travel and migration from countries within the European Union where *T. solium* is endemic [16,17].

This study has limitations. First, native stool samples were sent by mail to our laboratory, which may have affected the detection of pathogens. This particularly applies to *Shigella dysenteriae* and *S. boydii*, which are known to be more sensitive to environmental stress than other *Shigella* spp. Besides, the estimated meta-analytic sensitivities of stool microscopy of one vs three samples (Kato-Katz) are, for *A. lumbricoides* 63.8 vs 70.4%, *T. trichiura* 82.2 vs 90.5%, and hookworm 59.5 vs 74.3% [18]. Thus, the presented data are likely to underestimate the prevalence of enteric pathogen carriage in newly arrived asylum seekers to a certain extent. These differences, however, are not large enough to invalidate our conclusions. Second, during the peak of the refugee crisis, most reception centres were providing food through catering services. In settings where asylum seekers are involved in food handling, stool screening for enteric pathogens needs to be evaluated differently. Finally, although of interest in the given context, we were not able to report on microscopy results of protozoa (e.g. *Giardia lamblia*, *Entamoeba histolytica*), as these are not part of the mandatory screening. However, in light of evidence on its person-to-person transmissibility from research in day care centres [19,20], we acknowledge that screening for *G. lamblia* may be a meaningful addition to the proposed screening in children.

Critically reviewing the risk assessment above and acknowledging the limitations of our study, we conclude that routine screening of newly arrived asylums seekers for enteric bacteria with the aim to prevent onward transmission in the described population and setting is obsolete. Similar reasoning applies for most helminth infections currently screened for in Rhineland-Palatinate. At the same time, our data demonstrate that surveillance of imported enteric bacteria and parasites provides an important basis to identify particular individual and public health risks. To prevent severe, long-term morbidity due to schistosomiasis and cysticercosis and the further spread of these infections in Europe, we recommend screening of Eritrean asylum seekers for helminth infestation with a focus on *Schistosoma* and *Taenia* spp., using targeted methods at specialised institutions. Further research is needed to clarify the endemicity of *T. solium* in Eritrea [16].

We recommend continuing the screening of one stool sample of asymptomatic children and adolescents for enteric pathogens. The prevalence of enteric pathogens was elevated in these groups that are also less likely to adhere to hand hygiene and other individual infection prevention measures. Besides, children are known to suffer most from the harmful consequences some of the helminth infestations may have, such as anaemia, stunting, and nutrient deficiency [21-23]. Hence, targeted screening of this risk group would allow to prevent such harm, both from an individual and public health point of view. It should also allow future identification of risk groups among children that may require intensified screening at specialised institutions. Similarly, ongoing testing for enteric pathogens in a representative subsample of all newly arrived asylum seekers will be required to adapt the targeted approach to changing patterns of migration and associated risks.

## Note

*This designation is without prejudice to positions on status, and is in line with United Nations Security Council Resolution 1244/99 and the International Court of Justice Opinion on the Kosovo declaration of independence.
